# Determination of the fate of Cholecalciferol injected by the basis of 25D3 plasma concentration

**DOI:** 10.1038/s41598-023-29870-9

**Published:** 2023-03-11

**Authors:** Seyed Ali Mousavi Rad, Mohammad Rahim Haji Hajikolaei, Mohammad Nouri, Masoud Reza Seyfi Abad Shapouri, Masoud Ghorbanpoor

**Affiliations:** 1 Department of Clinical Sciences, Faculty of Veterinary Medicine, Shahid Chamran University of Ahvaz, Ahvaz, Iran; 2 Department of Pathobiology, Faculty of Veterinary Medicine, Shahid Chamran University of Ahvaz, Ahvaz, Iran; 3 Present Address: Department of Pathobiology, Faculty of Veterinary Medicine, Shahrekord University, Shahrekord, Iran

**Keywords:** Biochemistry, Physiology, Endocrinology

## Abstract

Sun exposure in bovines is believed to be the most important route of 25D_3_ synthesis in suitable latitudes. In some situations, e.g. breeding systems, solar radiation cannot reach or penetrate into the skin and thus causes the 25D_3_ deficiency. Because of the critical effect of vitamin D on the immune and endocrine systems, the plasma must be enriched with 25D_3_ in a short period of time. In such a condition, injection of Cholecalciferol has been recommended. However, to our knowledge, the certain dose of Cholecalciferol injection for rapid 25D_3_ plasma enrichment has not been verified. On the other hand, it seems that the basis 25D_3_ concentration can influence or shift the 25D_3_ metabolism at the injection time. In the same line, the present study, designed to induce the different basis 25D_3_ concentration in treatment groups, aimed at investigating the effect of Cholecalciferol intramuscularly injection with the intermediate dose (11,000 IU/kg) on the calves' plasma 25D_3_ with different basis 25D_3_. Besides, an attempt was made to clarify the time that 25D_3_ reaches the sufficient concentration after injection in different treatment groups. To do this, twenty calves of 3 to 4 months old were chosen for the farm with semi-industrial elements. Furthermore, the effect of optional sun exposure/deprivation and Cholecalciferol injection on the 25D_3_ concentration variations was assayed. To do this, the calves were divided into four groups. Groups A and B were unconstrained to choose sun to expose or shadow in a semi-roofed place, but groups C and D were restricted to the completely dark barn. The interference of the digestive system in supplying vitamin D was minimized through dietary. All groups had a different basic concentration (25D_3_) on the day 21 of the experiment. At this time, groups A and C received the intermediate dose of (11,000 IU/kg) Cholecalciferol intramuscularly (IM). After Cholecalciferol injection, the effects of basis 25D_3_ concentration on the details of variation and fate of plasma concentration of 25D_3_ were investigated. The data collected from the two groups C and D showed that sun deprivation without any vitamin D supplementation, could rapidly and severely deplete the plasma from 25D_3_. Cholecalciferol injection could not immediately increase the 25D_3_ in the groups C and A. However, this injection enriches the 25D_3_ to sufficient value after two weeks if the basis 25D_3_ of plasma is insufficient, i.e. less than 30 ng/mL. Moreover, the injection of Cholecalciferol could not significantly increase the 25D_3_ concentration in the group A that had a sufficient basis 25D_3_ concentration. Therefore, it is concluded that the variation of 25D_3_ in plasma, after injection of Cholecalciferol, depends on its basic level at the time of injection.

## Introduction

There are different sources of vitamin D in mammals. For instance, producing endogens synthesis from sun light or digestive route (exogenous route), particularly most of the herbivorous mammals can synthesize vitamin D from 7-Dehydrocholesterol (7-DHC) under the sun light exposure^1–5^, since most of the herbivores, bovines depend on sunlight to synthesize 25-Hydroxyvitamin D ( 25D_3_). Although 25D_3_ is neither the active form nor the final pathway of the metabolism of vitamin D, it is a routine task to measure 25D_3_ for vitamin D_3_ evaluation of plasma, because 25D_3_ half-life in plasma is long and it has a more stable structure compared to active metabolite of vitamin D (1,25-Dehydroxyvitamin D). When bovine skin exposes to UVB in proper latitude and appropriate angle of sun light, the 7-DHC changes to Vitamin D_3_^6^. Although most parts of the body in cows are covered by hair, the skin can efficiently synthesize vitamin D3^4^. Variation levels of vitamin D have different effects on the function or physiological activity not only in different animal but in some organ in same animal. For example, It is evident that 10 ng/mL of 25D_3_ is sufficient for Ca homeostasis and bone formation to prevent rickets and bone disorders in lambs^7^, llamas and alpacas^8^. However, it has been asserted that maintaining a physiological balance of calcium and phosphorus in cattle requires plasma concentrations of 25(OH)D larger than 20 ng/ml^9^. The perception for vitamin D optimum concentration changes after verifying the existence of vitamin D receptor nearly in all cells and its vital role in immune system signals and performance^10^. It is now confirmed that optimum concentration of 25D_3_ for proper performance of immune system and endocrine pathway, such as Insulin releasing, is approximately 30 ng/mL^11^. The bovine remains to be deprived of sunlight during the winter months in high latitude zones, in areas with overcast or dirty skies, and also in confined or roofed systems for breeding^6,12,13^.

In Iran, the main reason for the lack of vitamin D in bovines is insufficient UV light penetration to convert 7-DHC to vitamin D_3_ at sun striking angles less than 35 degrees, as it occurs in winter^6^. In these situations, in the breeding systems that cannot provide a proper dietary vitamin D regimen, it has been recommended that Cholecalciferol injection with 500–2000 IU/kg can prepare a sufficient concentration of 25D_3_ in ruminants^14^. However, this recommended dose cannot make a suitable concentration of 25D_3_ in all cows with different metabolic situations^15^. The injection of a high dose of Cholecalciferol causes the 25D_3_ increase with a week lag time^16^. Nonetheless, the lack of experimental studies focusing on the following points can be observed:

- Specific assay of the sunlight affecting the 25D_3_ synthesis in calves via maximum limitation of other sources of the precursor of 25D_3_, especially dietary route.
- Comparison of 25D_3_ concentration changes in groups receiving injectable Cholecalciferol and sunlight regimen.
- Assay the ability of the intermediate dose (11,000 IU/kg) of injectable Cholecalciferol to increase 25D_3_ concentration.
- Evaluation of changes in plasma 25D3 by measuring it in short time intervals (one week).
- Examining the effects of basic plasma 25D_3_ concentrations in its variation after Cholecalciferol injection with the intermediate dose in the calves.

Based on the above-mentioned points, the present study has been designed to induce the different basis 25D_3_ of plasma and evaluate the details of plasma 25D_3_ variations via measuring 25D_3_ in short time intervals during the inducing period of different basis concentration of 25D_3_ and after it. Another target was the observation of the ability of the 11,000 IU/kg Cholecalciferol intramuscular injection to change the 25D_3_ concentration. In all, the current study investigates the effect of the basic concentration of 25D_3_ on its variation after injection of intermediate dose of Cholecalciferol.

## Results

**Figure 1:**
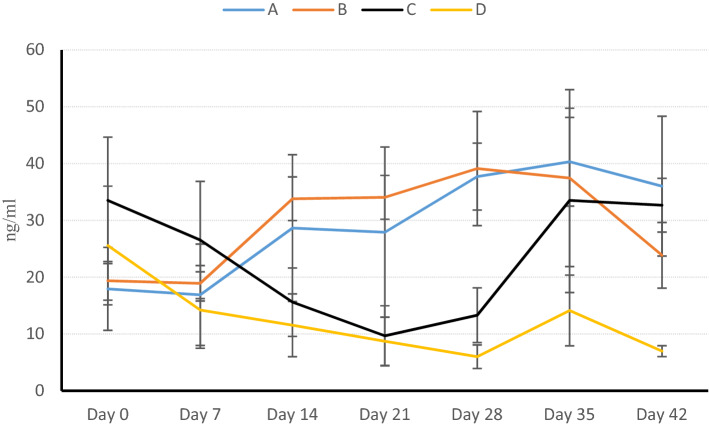
The 25D_3_ variations in six weeks of experiment. Day 21 was the injection time of vitamin D injection in groups A and C. The procedure of variations in the six weeks in group B was increasing (*P* < 0.05). The procedure of variations in the six weeks in group D was decreasing (*P* < 0.01). The 25D3 concentration after the third week was not significantly different between groups A and B. however, it was significantly different between C and D (*P* < 0.05). The procedure of variations was significant in all groups (*P* < 0.05) except A day 21.

The mean value of vitamin D variation in treatment groups during 42 days (six weeks) is shown in both Fig. 1 and Table 1. As mentioned earlier, day 21 was the fundamental day for Cholecalciferol injection for the members of A and C groups. For a better and more reasonable comparison, the results in the two timespans, before (from the beginning of the experiment to the day 21) and after Cholecalciferol injection (from day 21 to 42; the end of experiment), have been investigated. However, in the review of variations of groups, B and D are assumed as the two groups that have not had Cholecalciferol injection. Therefore, the groups B and D can be compared as treatment and control groups throughout the experiment.

**Table 1:** The amount of 25D_3_ (ng/mL) in each calf and Mean ± SD of variations of 25D_3_ (ng/mL) in all groups.

Group	Days of the sampling							
	Case number	Day zero	Day 7	Day 14	Day 21*	Day28	Day 35	Day42
A	318	8.4	3.1	10.4	9.3	28.7	29.7	24.3
	304	24.6	25.2	41.8	44.5	44.2	51.1	50
	308	11.8	13	20.3	14.9	37.8	40.5	48.7
	341	23.6	22.8	37.1	36	36.3	37.3	30.2
	310	21.3	20.4	33.6	34.9	41.5	43	26.9
Mean		17.94	16.9	28.64	27.92	37.7	40.32	36.02
SD		7.31	8.92	12.91	14.99	5.89	7.81	12.31
B^‡^	314	23.4	22.5	31.3	38.3	38.7	64.1	33.7
	315	14.6	13.9	29.1	30.5	28.6	28.4	21
	349	21.1	19.5	34.5	30.7	55.2	35.3	23.6
	338	20.4	18.7	34.9	32.7	33.1	25.6	18.7
	312	17.3	20	39.2	38.1	40	33.8	22.2
Mean		19.36	18.92	33.8	34.06	39.12	37.44	23.84
SD		3.41	3.12	3.84	3.86	10.04	15.57	5.78
C^†^	300	35.4	31.2	18.8	7.4	9.4	20	27.2
	302	44.5	34.9	18.8	18.3	20.9	59.6	39.1
	303	35.1	29.1	19.8	9.2	10.8	23.6	35.1
	326	37.9	29	15.4	9.6	10.1	39	29
	342	14.7	8.5	5.2	3.9	15.3	25.4	32.9
Mean		33.52	26.54	15.6	9.68	13.3	33.52	32.66
SD		11.13	10.32	6.02	5.29	4.82	16.21	4.73
D^‡^	313	18.4	9.1	6.5	7.4	8	9.1	6.2
	348	31.4	11.1	13.1	14.8	6.1	10.7	6.4
	309	37.5	24.5	16.2	10.1	8	24.2	8.6
	299	11.5	8.8	4.9	3	3.2	10.5	6.6
	301	29.1	17.6	17	8.3	4.7	16.2	7.1
Mean		25.58	14.22	11.54	8.72	6	14.14	6.98
SD		10.44	6.73	5.53	4.26	2.07	6.22	0.96

Groups A and B: were free to choose sun or shade. Group C and D: were restricted in the completely dark barn. Number of cases = The number of ear plaque of examined calves. SD = Standard Deviation of Mean. The day 21 (the fourth week of the experiment) was the date when groups A and C received 11,000 IU/kg of injectable Cholecalciferol.

*The mean of variations of groups A and B had significant different with groups C and D after four weeks (*P* < 0.01).

^†^The procedure of variations in the six weeks in group D was decreasing (*P* < 0.01).

^‡^The mean of 25D_3_ in groups B and D were 36.02 and 6.98 ng/mL respectively in six weeks of experiment.

### 25D_3_ variations before Cholecalciferol injection

**Figure 2:**
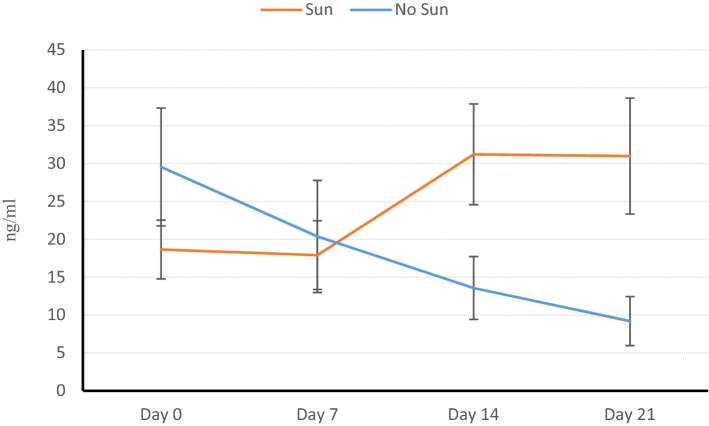
The effects of sun on 25D_3_. The 25D_3_ Variations in groups A + B (exposed to sun) and C + D (Non-exposed to sun) before Cholecalciferol injection (from Day zero to Day 21).

Although these calves were all from the same herd and were randomly selected and divided into 4 groups, due to unknown reasons, their basic vitamin 25D_3_ levels were different from each other on the first day of the experiment. 25D_3_ variations in groups A and B (A + B), as the group exposed to the sunlight and groups C and D (C + D), as the other group having been deprived of the sunlight during the first 21 days of the experiment were investigated. As shown in Table 2 and Fig. 2, 25D3 plasma concentration of group A + B increased to sufficient value from insufficient concentration at the beginning of the experiment during 21 days (*P* < 0.001). In group C + D, as the sunlight deprivation group, the 25D3 depleted from plasma significantly during 21 days (*P* < 0.001).

**Table 2:** The Mean ± SD of 25D_3_ in groups A + B and C + D before Cholecalciferol injection.

Groups	Days of the sampling			
	Day zero	Day 7	Day 14	Day 21*
A + B				
Mean	18.65	17.91	31.22	30.99
SD	3.88	4.53	6.65	7.65
C + D				
Mean	29.55	20.38	13.57	9.2
SD	7.78	7.4	4.15	3.23

The 25D3 in Group A + B increased by 12.34 ng/mL and decreased by 20.35 in groups C + D. The time factor had a significant effect on 25D3 variations in both groups (*P* < 0.01). The interaction between time and group was significant (*P* < 0.001).

### 25D_3_ variation after Cholecalciferol injection

Investigating the four treatment groups from the third week of the experiment on shows a significant relationship between the time factor as well as being in the different treatment groups and the 25D_3_ concentration (*P* < 0.001). Moreover, both the effectiveness of different kinds of treatment groups and the time interval were significant (*P* < 0.01).

The process of 25D_3_ change in groups A (sunlight and Cholecalciferol injection) and B (sunlight) relative to each other was not significant (*P* > 0.05); Nevertheless, the variation of 25D_3_ in group A differed significantly from the members of groups C (*P* < 0.01) and D (*P* < 0.001). Furthermore, the variations of 25D_3_ in groups B, C, and D were significant (*P* < 0.01). Investigation of groups A and C, as the two groups that received vitamin D, showed that injection of Cholecalciferol enhances the 25D_3_ concentration in both groups. However, this enhancement was only significant in group C (*P* < 0.001).

### Groups B and D

Analyzing the variance with repeated measures in groups B and D, i.e. the two groups that did not receive vitamin D, showed a significant variation in 25D_3_ concentration at the end of the experiment (*P* ≤ 0.05).

In group B, the 25D_3_ concentration in the day14 had a significant difference compared with that of the day zero, Moreover It has been without any significant change until the day 35. However, it changed significantly towards the end of the experiment (*P* ≤ 0.05).

In group D, concentration of 25D_3_ in the first week had a significant difference with all other weeks (*P* ≤ 0.05). Furthermore, 25D_3_ concentration in the second week was different, in terms of value, with the fourth and sixth week significantly (*P* ≤ 0.01). Such a substantial difference was seen in the fourth week compared with the fifth week as well as the fifth week with the sixth week.

## Discussion

According to the results of the present study, 25D_3_ concentration decreases rapidly and severely during four weeks if ruminant calves are deprived of the sunlight. Due to the obtained statistical results, 25D_3_ concentration decreased to below 10 ng/mL which is in agreement with the previous studies^4,17–20^. These calves accessed to a sort of diet containing 20% dry hay (Alpha- alpha) dried by the sun. The sun dried Alpha-alpha contains nearly 2500 IU/kg of vitamin D_2_^21^. However, this value of vitamin D_2_ is negligible in the ruminant diet concerning its catabolism to 10-keto-19-nor-Vitamin D_2_ in the rumen^22^. Therefore, the minimum participation of the alimentary system in vitamin D preparation is assured.

Casas et al. (2015)^20^ observed a severe decrease in 25D_3_ concentration in autumn weaned calf’s experience; yet, calves received 800 IU/day of Cholecalciferol. After measuring 25D_3_ every three months, they reported a general variation of 25D_3_ after each season but the important details of the increase and/or decrease of 25D_3_ in different seasons were not investigated in their study. Nelson et al. (2016)^13^ and Krueger et al. (2014)^23^ observed the plasma 25D_3_ reduction to be less than 15 mg/mL in calves deprived of the sunlight and cholecalciferol supplement in 5 weeks. The 25D_3_ concentration in our study decreased from 30 to less than 10 ng/mL after four weeks of starting the experiment in the defined groups of C and D. Moreover, in contrast to the aforementioned studies, plasma 25D_3_ decreased to below 10 ng/mL in all participants of groups C and D. Most likely, because the calves in groups C and D were confined in a completely dark place accompanied by a diet with low quality and low Alpha-Alpha percentage, the plasma experienced a severe depletion of 25D_3_ with a short duration (four weeks) compared to Nelson's and Krueger’s experiments^13,23^.

As mentioned earlier in this the study, we randomly divided the participants into four groups of A, B, C, and D to create different basic 25D_3_ by 26-Aug. Group D calves remained in a dark place without any vitamin D regimen until the end of the experiment. The mean value variation of 25D_3_ in group D had a descending variation until 2-Sep. However, it increased significantly during one week from 2-Sep to 9-Sep. Yet, the reason is unclear to us. It could be assumed assumed that this was caused by non-compliance with the ratio of straw and hay in favor of hay by the workers who made the TMR, so, this mistake was making the increase in the 25D_2_ concentration, not 25D_3_. But the general variation of 25D_3_ in group D was significant. Hymøller and Jensen (2010)^4^ also kept the dairy cows in the barn for six months. They assayed 25D_3_ variations every five days at a 30-day duration which were in agreement with our results in group D.

In groups A and B, we confirmed that letting calves free to choose either light or shade in an environment that had both choices during the day could raise 25D_3_ above 30 ng/mL during two weeks. This was not seen by the researchers after deeply reviewing the relevant literature available to this date. In the previous studies, the members of the treatment groups were free in pastures with no access to places with constant shade or at least these places were not mentioned by the researchers^4,13,18–20^.

In the end of third week of the experiment, Cholecalciferol (11,000 IU/Kg) was injected intramuscularly in groups A and C. As an interesting observation, no significant 25D_3_ difference was seen between groups A and B, i.e. the two sun-exposed groups. Hidiroglou et al. (1979)^12^ in Canada and Hymøller et al. (2009)^19^ in Denmark designed separate and almost similar studies and achieved the results similar to those of the present study. They also established a different concentration of 25D_3_ in two groups of cows stored in roofed places by different vitamin D regimens. After the transition of the cows to pasture, the difference of 25D_3_ concentration between groups was removed over time, whereas, the former vitamin D regimen in each group was maintained^12,19^. In the aforementioned experiments and those conducted in the current study lack of increase in 25D_3_ represents the existence of a regulatory mechanism. This mechanism prevents the over increase of 25D_3_ concentration when cows are exposed to sunlight sufficiently as well as using supplemented or injectable Cholecalciferol. Some comments and experiments have been made to justify this phenomenon. After weekly exposure to UVB in humans, Bogh et al. (2012)^24^ observed a significant rise in 25D_3_, but the value of increase in 25D_3_ gradually decreased with time in weekly exposure, indicating a condition of saturation in vitamin D synthesis. They concluded that if basis 25D_3_ in plasma is in sufficient concentrations, the 25D_3_ inhibits the 25-hydroxylase in the liver and decreases the enzyme activity.

Probably this phenomenon caused concentration of 25D_3_ in the participant to show a smaller increase range after each exposure to UVB. The calves in group A had a sufficient 25D_3_ concentration in Cholecalciferol injection time. Although we could not measure the 25-hydroxylase, we could suppose this feedback mechanism triggered in group A was because of a sufficient concentration of 25D_3_ due to the sun exposure which prevented the 25D_3_ increase^24^. In an interesting observation, vitamin D was added to the panther chameleon's diet. The vitamin D supplemented group exposed themselves to UVB lesser than the control group^25^. Unfortunately, we did not monitor calves of group A to distinguish their selective behavior between sun and shade after Cholecalciferol injection. However, it was concluded that both panther chameleon and cow depended on UVB and endogenous synthesis of vitamin D^4,19,25^. It could probably be concluded that this phenomenon happened and the participants of group A exposed themselves less to the sunlight after Cholecalciferol injection. Probably it can be assumed, in the present study, that the entrance of a sufficient amount of Cholecalciferol triggers negative feedback that decreases the precursor of 25D_3_ on the skin surface and maintains the 25D_3_ concentration constant. However, due to the lack of information about the relationship between Cholecalciferol and 7-dihydroclosterol, we propose an experiment to investigate the relationship between injection of Cholecalciferol and 7-dihydroclosterol in the skin. It should be kept in mind that the absorption of vitamin D is passive; so, there is little chance to conclude that the absorption of vitamin D from the intestine is reduced by increasing the precursor 25D3 in plasma^19^. there is a slight chance to conclude that by increase precursor of 25D3 in plasma, Vitamin D absorption decreases from the intestine19In general, cows are likely able to maintain a constant concentration of 25D_3_ with one or a combination of methods.

Generally, the issue of plasma 25D_3_ fluctuations under sunlight and shadow or dark place is in line with some other studies^12,18–20^. Indeed, aforementioned studies, in line with our observation, confirm that bovines depend on the endogenous source of vitamin D. Yet, 25D_3_ cannot be saved in the calves’ body for as long as four weeks.

Contrary to group A, Group B did not receive vitamin D (Cholecalciferol) injection. The only source of the vitamin D regimen in group B was the option of using sunlight as their own choice. Group B reached approximately 40 ng/mL until fourth week of experiment. Hymøller, and Jensen (2010)^4^ observed the same results in their study. In another experimental study by Nelson et al. (2012)^11^, newborn calves were sun-exposed alone and they had not vitamin D regiment; therefore, 25D_3_ increased to nearly 30 ng/mL from under 10 ng/mL in four weeks duration.

Group C had a low basis 25D_3_ concentration after three weeks’ limitation in the dark place. After Cholecalciferol injection, 25D_3_ started to increase during the first week after injection (fourth week of the experiment). However, this increase was not significant. As it is observed in Fig. 1, the 25D_3_ concentration increased significantly in plasma with a steep slope and significantly after the fourth week of the experiment or two weeks after vitamin D injection which is in turn in agreement with Bogh et al.’s theory^24^. In fact, low plasma 25D_3_ concentration on the injection day or low plasma's basis 25D_3_ caused the 25-hydroxylase to stay active and thus fulfilled its duty. It should be noted the 25D_3_ concentration did not increase significantly immediately after Cholecalciferol injection in group C. It took approximately two weeks to be significant. Hollis et al.’s theory (1977)^16^ may explain the reason why the 25D_3_ concentration did not increase directly after Cholecalciferol injection. They injected two-fold (22,000 IU/kg) of Cholecalciferol to adult dairy cows. They also did not see a significant increase in 25D_3_ concentration in the first week after injection. They assumed that when a high dose of Cholecalciferol enters the body, the physiologic feedback starts to decrease the activity of P450 enzymes (25-hydroxilase). This feedback probably was the reason for the insignificant increase of 25D_3_ in plasma during the first week after injection. Krueger et al. (2014)^23^ injected 800 IU/kg Cholecalciferol into newborn calves. In contrast to the observations made by both Hollis^16^ and present study, the concentration of 25D_3_ increased two folds and significantly during one week after injection. In their study, the low basic concentration of 25D_3_ on the injection day and the low dose of Cholecalciferol that was injected are noticeable. Based on the above-mentioned details, we can assume that to induce the fast increase of plasma 25D_3_, it is more logical that the minimum advised dose be prescribed and basis 25D_3_concentration be considered. In contrast with group C, the plasma concentration of 25D_3_in group D kept decreasing after third week of the experiment to 6.98 (ng/mL) at the end of experiment. At day 21, concentrations of 25D_3_ was almost equal in groups C and D, but after seven days, 25D_3_ concentrations were 13.3 and 6 ng/mL, respectively. These values reached 33.52 and 14.14 in the fifth week (two weeks after Cholecalciferol injection), respectively and the difference in the 25D_3_ concentration between these two groups was significant in this time.

## Conclusion

Sunlight with an appropriate wavelength is a major and free source for 25D_3_ metabolism via the endogenous route in bovines. Giving cows the freedom to choose sun or shade in semi roofed places can raise the 25D_3_ to sufficient concentration within 2 to 3 weeks. Cows were deprived of sunlight UV exposure by roofed places experience severe hypovitaminosis D during 2 weeks, if the owners do not provide other sources of vitamin D. Cholecalciferol injection is an important method for curing hypovitaminosis D. The intermediate dose of Cholecalciferol IM injection probably cannot raise the plasma 25D_3_ less than 14 days. The basic concentration of 25D_3_ has a key role in determining the effect of Cholecalciferol injection on the 25D3 plasma concentration.

## Material and methods

### Animals selection, housing, and separate the treatment groups

Twenty calves were selected in the semi industrial farm in Iran, Qazvin province. All of the animals were 3 to 4 months old and were not drinker. Because of the underprivileged financial situation of the farm owner, the calves' diet was not nourished properly; so, it consisted of 20% Alfalfa and 80% straw without any vitamin and minerals supplement. This situation was quite suitable for our study, because we could measure the sunlight effect or Cholecalciferol injection on the 25D_3_ metabolism with minimum interference of the digestive system.

Calves were separated randomly into four groups: Groups A and B: these groups were kept in the semi-roofed place. This housing system was made in such a way that group members could freely move and get exposed to the sun if they liked to. Groups C and D: in these groups, calves of these groups were kept in a restricted place. The barns brick roof and walls did not allow the sunlight to penetrate. This housing system made the barn dark with no light inside. Moreover, no electric lighting system was used to prevent vitamin D_3_ production by UV light of lamps.

### Blood sampling

The zero time was set as the calves were divided into four groups. Moreover, the blood samples were collected from each participant for evaluation of desired parameters at this time. The sampling procedure was repeated weekly for six weeks. Blood samples were collected by gel activator vacuum tube from the jugular vein after aseptic. All collected samples were kept in the cool box at 4 °C during the sampling. Serums were separated by centrifuge and stored in -20 °C until the test.

### Vitamin D measurement and injection

At the beginning of the fourth week of the experiment, the calves of groups A and C were injected with 11,000 IU/kg Cholecalciferol IM. The level of sera 25D_3_ were measured by a commercial competitive ELISA kit (Ideal Tashkis, Iran) from the first day of the experiment and this process was weekly repeated. Based on the information provided by the producing company, the kit was able to determine the amount of 25D_3_ in serum or plasma, in range of 2 to 120 ng/mL with a precision of ± 10%. Briefly of the procedure according to the instruction of the producing company; 25 µl of samples, calibrators and control were added in separate duplicate wells. After addition of 100 µl of Vit D releasing to all wells, Plate was incubated at room temperature for 30 min and then was washed five times. Then 100 µl of Vit D HRP conjugate were added into the wells and allowed to react for 30 min at room temperature. After washing as above, the wells were poured with 100 µl of chromogen substrate (TMB + H_2_O_2_) for about 20 min. Finally, the reaction was stopped by the addition of 50 µl of stope solution and Optical density (OD) of wells were read in an ELISA reader (Accureader, Taiwan) at a wavelength of 450 nm. The Vit D concentration of each samples were calculated based on standards with a 4 parameter logistic (4PL) software.

### Statistical analysis

In this study, to detect the main effects of treatment, time, and the interaction between treatment and time, the calves nested within the treatment group were assessed using two way repeated measure ANOVA analysis of variance and LSD post hoc. Due to the relevance of the data, the distribution of the difference in the data should follow the normal distribution. Therefore, before ANOVA the data were evaluated by Shapiro–Wilk test and so it was found that the distribution of the data was normal (*P* > 0.05). In all statistical analyses, the *P* value of less than 0.05 was considered significant.

### Ethical approval

This manuscript was extracted from thesis of Seyed Ali Mousavi Rad, a postgraduate student in the Faculty of Veterinary Medicine, Shahid Chamran University of Ahvaz. It has been approved by Ethics committee of Shahid Chamran University of Ahvaz and documented by number: EE/1401.2.24.140941/scu.ac.ir. All experiments were performed in accordance with the proposal approved by this committee. All the methods were carried out in accordance with relevant guidelines and regulations. All the experiments were carried out in accordance with ARRIVE guidelines. This study was carried out with the consent of the owner of the animals to use animals.

## Data Availability

All data analyzed during this study are included in this article.
